# Supplementary material for: 3D single cell scale anatomical map of sex-dependent variability of the rat intrinsic cardiac nervous system
Source: iScience. 2021 Jul 19;24(7):102795. doi: 10.1016/j.isci.2021.102795 (PMC8324857; doi:10.1016/j.isci.2021.102795)
Supplement: Document S1. Figures S1–S8 and Table S1 [file mmc1.pdf]

iScience, Volume *24*

Supplemental information

3D single cell scale anatomical map of sex-dependent variability of the rat intrinsic cardiac nervous system

Clara Leung, Shaina Robbins, Alison Moss, Maci Heal, Mahyar Osanlouy, Richard Christie, Navid Farahani, Corey Monteith, Jin Chen, Peter Hunter, Susan Tappan, Rajanikanth Vadigepalli, Zixi (Jack) Cheng, and James S. Schwaber

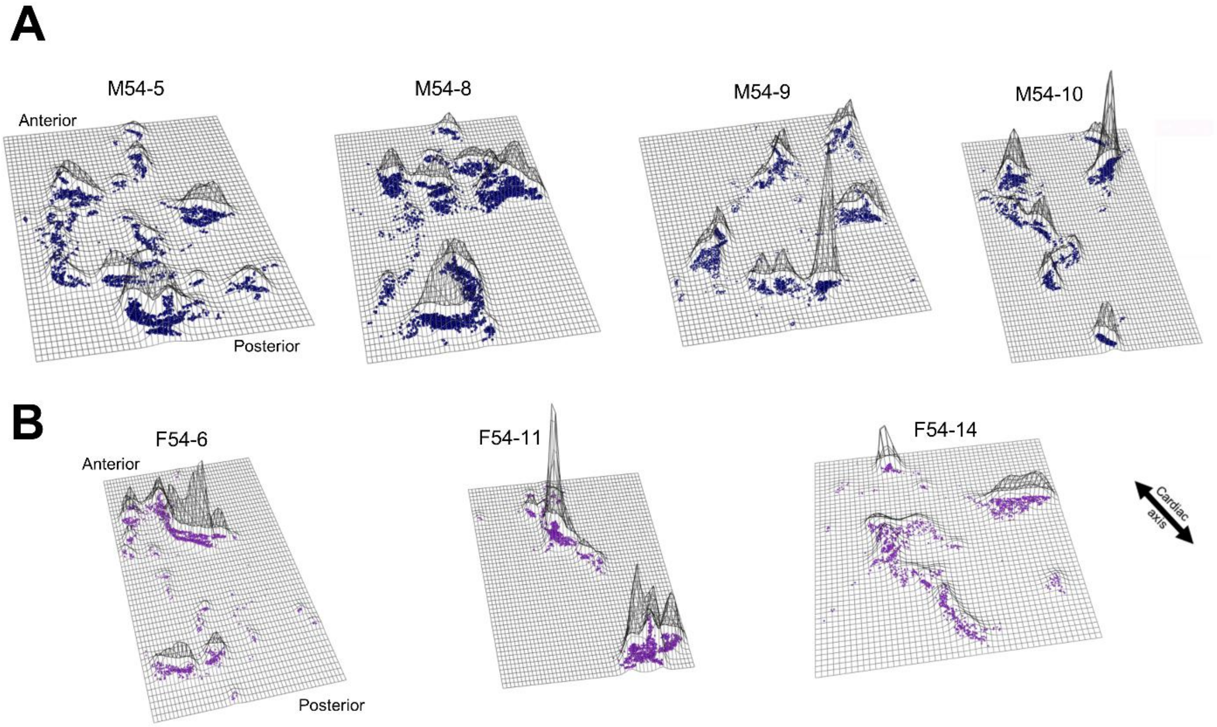

Figure S1: Flatmap projection showing distribution of neurons within the rat ICNS across individuals and sexes, related to Figures 2,7. (A,B) Flatmap projections derived from PCA plots are used to show the spatial data and packing density of the neurons in male (A) and female (B) ICNS. Each dot represents a single neuron. The anatomical orientation corresponding to the whole heart is indicated. Blue: males; Purple: females.

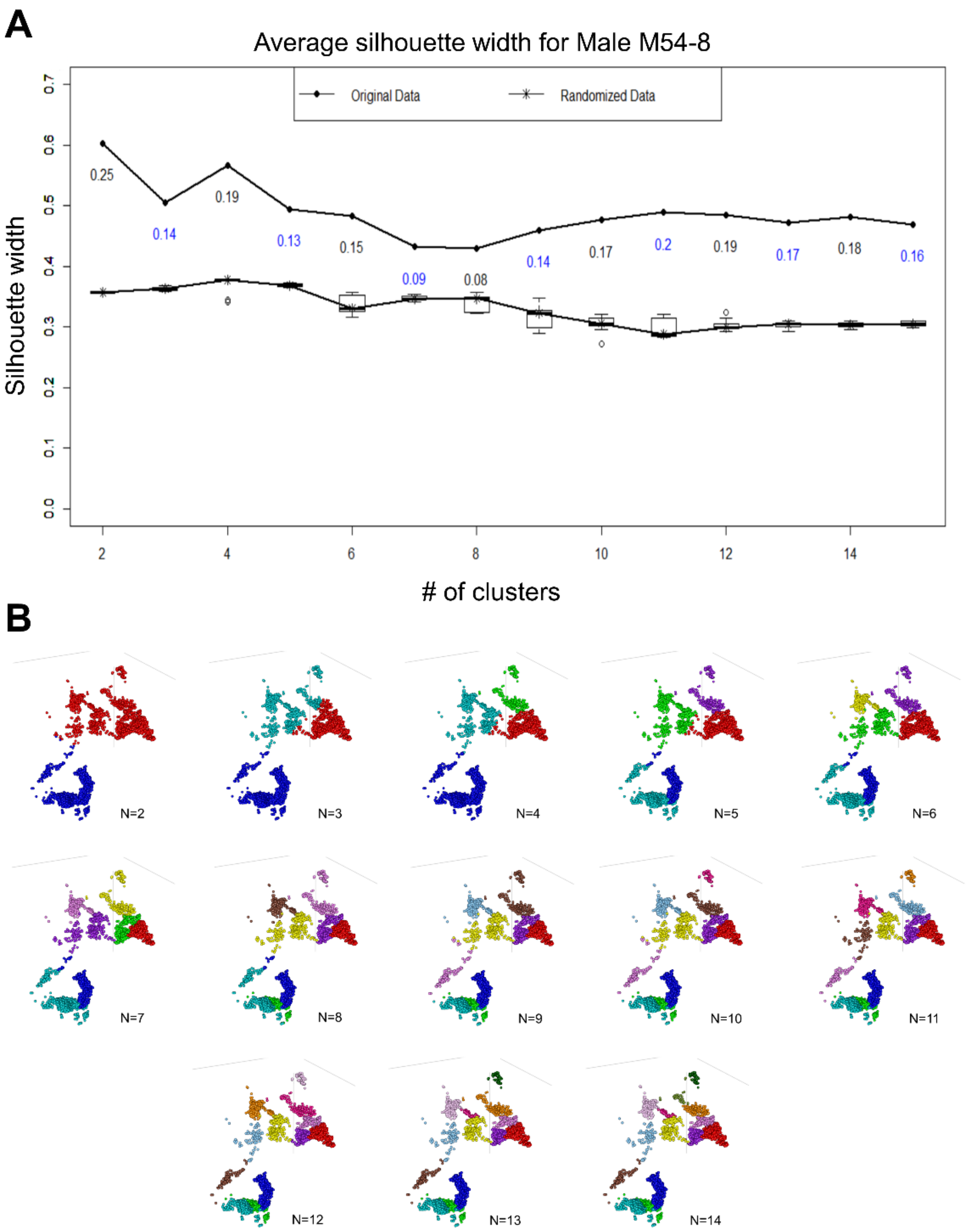

Figure S2: PAM analysis in male ICNS, related to Figure 3A; Figure 7C. Partitioning around medoids (PAM) analysis was performed on the ICNS coordinates for each cluster number, resulting in a silhouette coefficient describing how well the clustering fit the data. The coordinates were additionally randomized and subjected to the same analysis ten times to compare the silhouette width of randomized data to the original coordinates. **(A)** Graph comparing the silhouette width for both original and randomized data against the cluster number for male heart M54-8. The numbers below the line of original data in the plot shows the difference between the silhouette width of the original data and the mean of the randomized data. **(B)** For all possible cluster numbers, ICNS representations are colored based on the PAM assigned clustering.

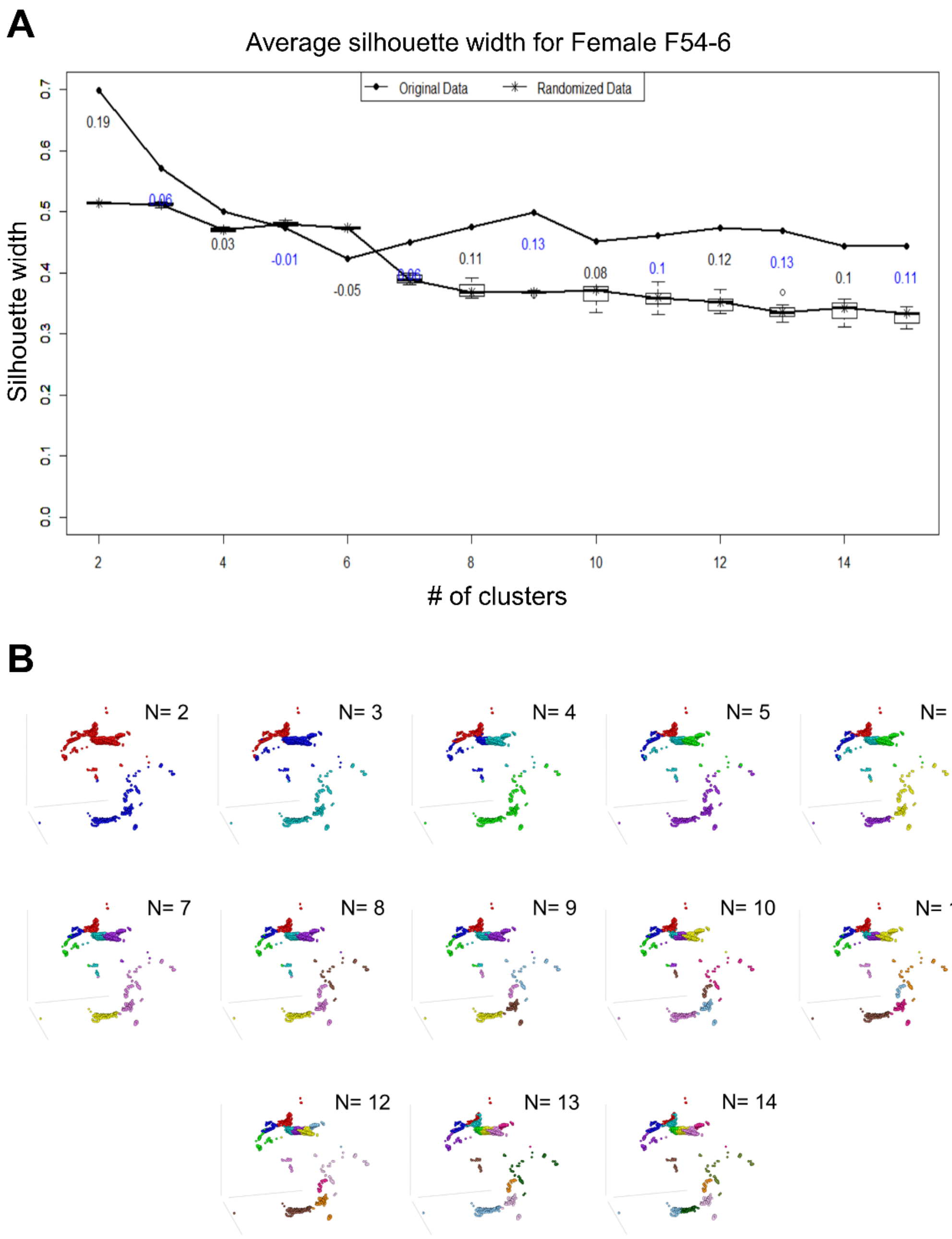

Figure S3: PAM analysis in female ICNS, related to Figure 7D. Partitioning around medoids (PAM) analysis was performed on the ICNS coordinates for each cluster number, resulting in a silhouette coefficient describing how well the clustering fit the data. The coordinates were additionally randomized and subjected to the same analysis ten times to compare the silhouette width of randomized data to the original coordinates. **(A)** Graph comparing the silhouette width for both original and randomized data against the cluster number for female heart F54-6. The numbers below the line of original data in the plot shows the difference between the silhouette width of the original data and the mean of the randomized data. **(B)** For all possible cluster numbers, ICNS representations are colored based on the PAM assigned clustering.

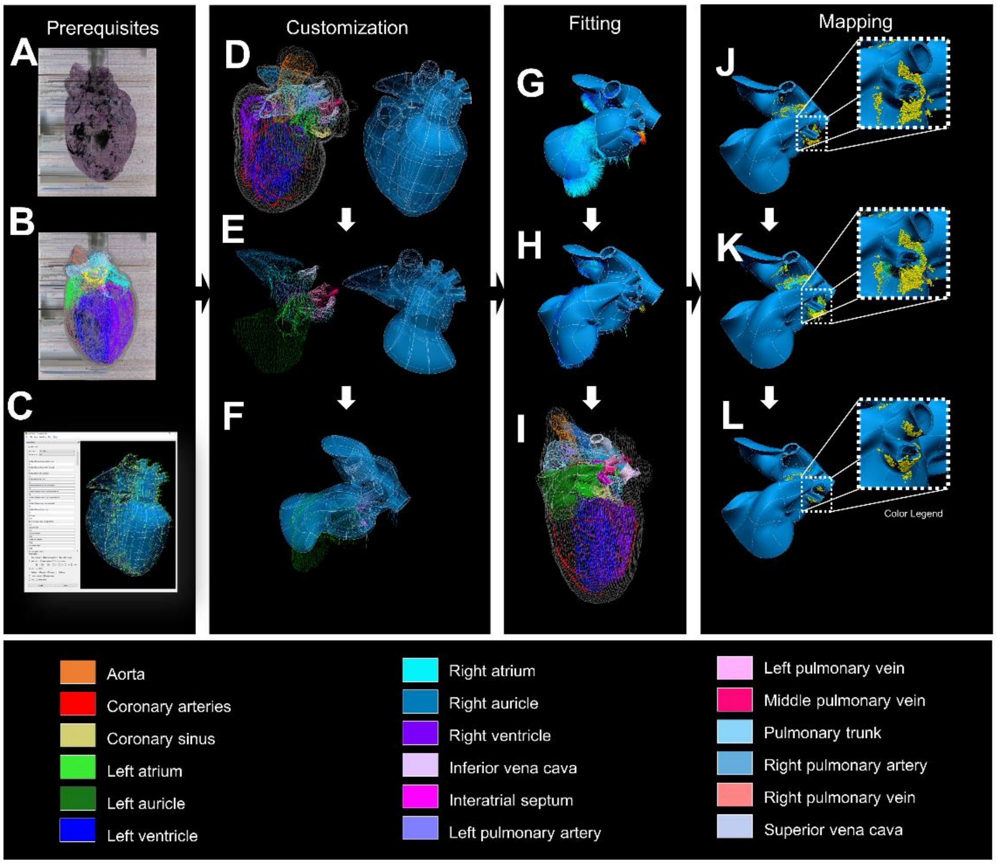

Figure S4: Scaffold fitting pipeline, related to Figure 6A, Video S3. (A-C) Three fundamental prerequisites are required for the mapping process. A volumetric image of the heart (A) is reconstructed to segment the regions into tracing contours (B). A corresponding scaffold is then generated from the Scaffold Maker software interface (C) to match the contour data. **(D-F)** Each heart must be customized to match the scaffold parameters to the data. The contoured heart data and the corresponding heart scaffold (D) are trimmed to include only the atria and auricles, pulmonary veins, superior vena cava, inferior vena cava, and interatrial septum (E). The scaffold is then aligned to the data through a rigid transformation process (translation, rotation, and scaling), and its parameters are tweaked empirically to provide the closest matching shape to the data (F). **(G-I)** the data must be fit to the scaffold and includes deformation of the scaffold to obtain an accurate shape of the data. Every point on the contour is projected onto the closest point on the surface of the scaffold (G) to find the sum of the Euclidean distances of these projections. This sum is then minimized through an iterative optimization process which deforms the scaffold to "fit" the data (H). The resulting fitted scaffold is superimposed on the original heart contour (I) for a further qualitative validation. **(J-L)** The ICNS data is then mapped onto the fitted scaffold. With the scaffold in the same coordinate system as the original data, the ICNS can be mapped onto the scaffold as element "material" coordinates. Some ICNS neurons are within the scaffold elements and others are wrapped around it (J). Those within preserve their exact spatial locations while the ones "hanging" around are projected to the nearest surface using a nearest search algorithm (K). This mapping results in embedding of ICNS into the scaffold and preserving their spatial distribution and cluster variation (L). Color legend shows the colors of every anatomically labelled region.

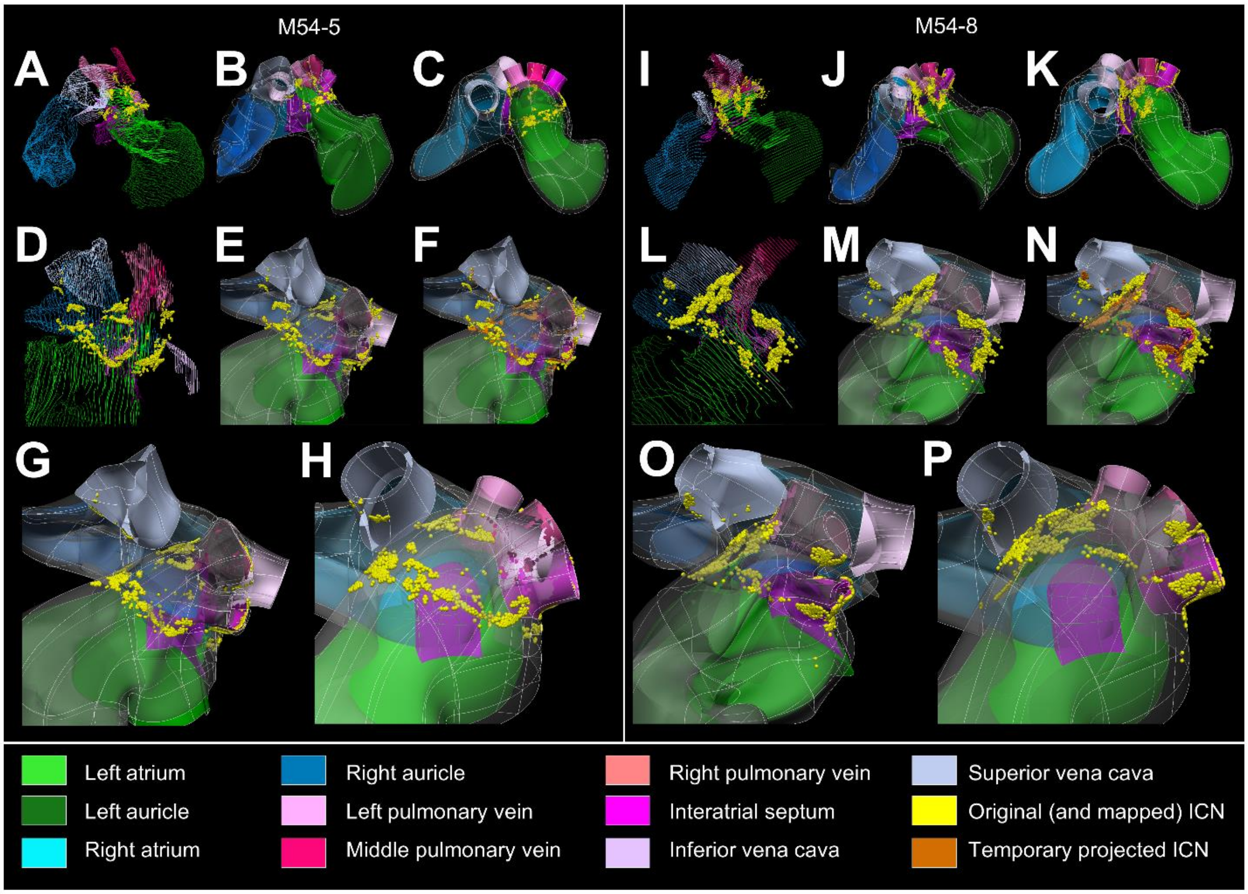

Figure S5: Mapping of ICNS from two male hearts onto the heart scaffold, related to Figure 6B,C, Video S3 . Mapping results for the two male rat hearts: 54-5 (A-H) and 54-8 (I-P). **(A-C,I-K)** Big-picture representations of the original contour data (A,I), fitted scaffold (B,J), and generic scaffold (C,K) with ICNS neurons shown in yellow spheres. ICNS data is mapped into the element material coordinates for (B,J) and (C,K). **(D-H,L-P)** Close-up, superior views from the left side of the data and scaffold. Distribution of ICNS neurons above the left atrium and around the pulmonary veins (D,L) and ICNS neurons transformed onto the corresponding fitted scaffolds (E,M) . Projection of ICNS onto the surface of the fitted scaffold (F,N) The [temporary] orange spheres represent the mapped locations of the original locations of the ICNS shown in yellow spheres. The exact location of any yellow sphere within the scaffold is preserved while for those yellow spheres located outside of the scaffold, an estimated location is found by projecting the ICNS neurons to the nearest surface plane. The resulting map of the ICNS data on the fitted (G,O) and generic scaffold (H,P). Note that the [temporary] orange color is changed back to yellow for consistency. The results show how this approach can provide the means of mapping and storing the spatial locations of the ICNS neurons within a quantifiable reference frame while keeping the pattern of ICNS distribution intact, and how this reference frame can provide means for comparing variation between different species.

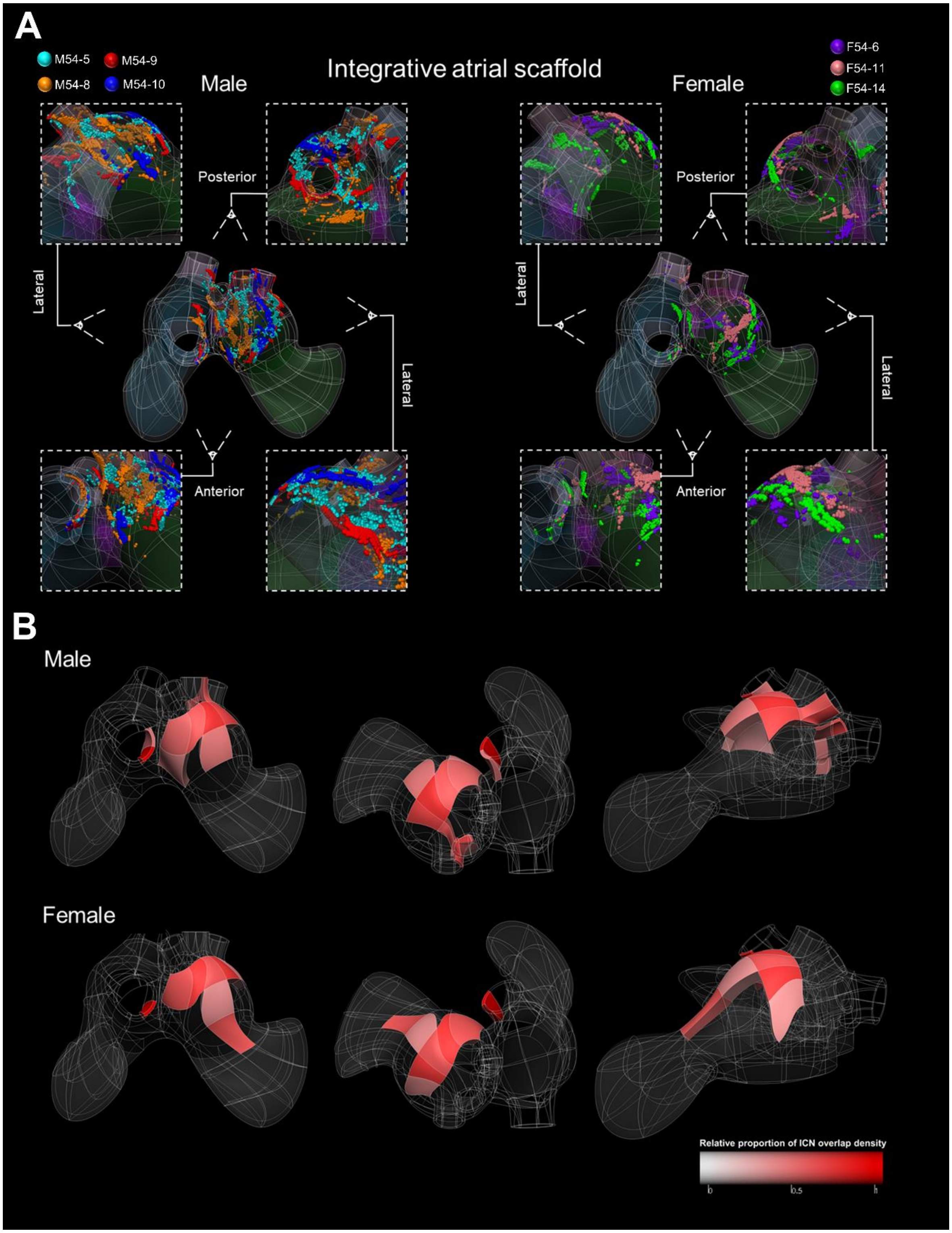

Figure S6: 3D scaffold comparing males to females, related to Figure 6D,E. (A) Integration of four data sets onto one generic scaffold for the male rat (left) and integration of three data sets onto one generic scaffold for the female rat (right). **(B)** Looking at the same regions of interest in figures 4 and 5, varying colors of pink are used to indicate the proportion of neuronal overlap between the four male datasets from (top) and the three female datasets (bottom).

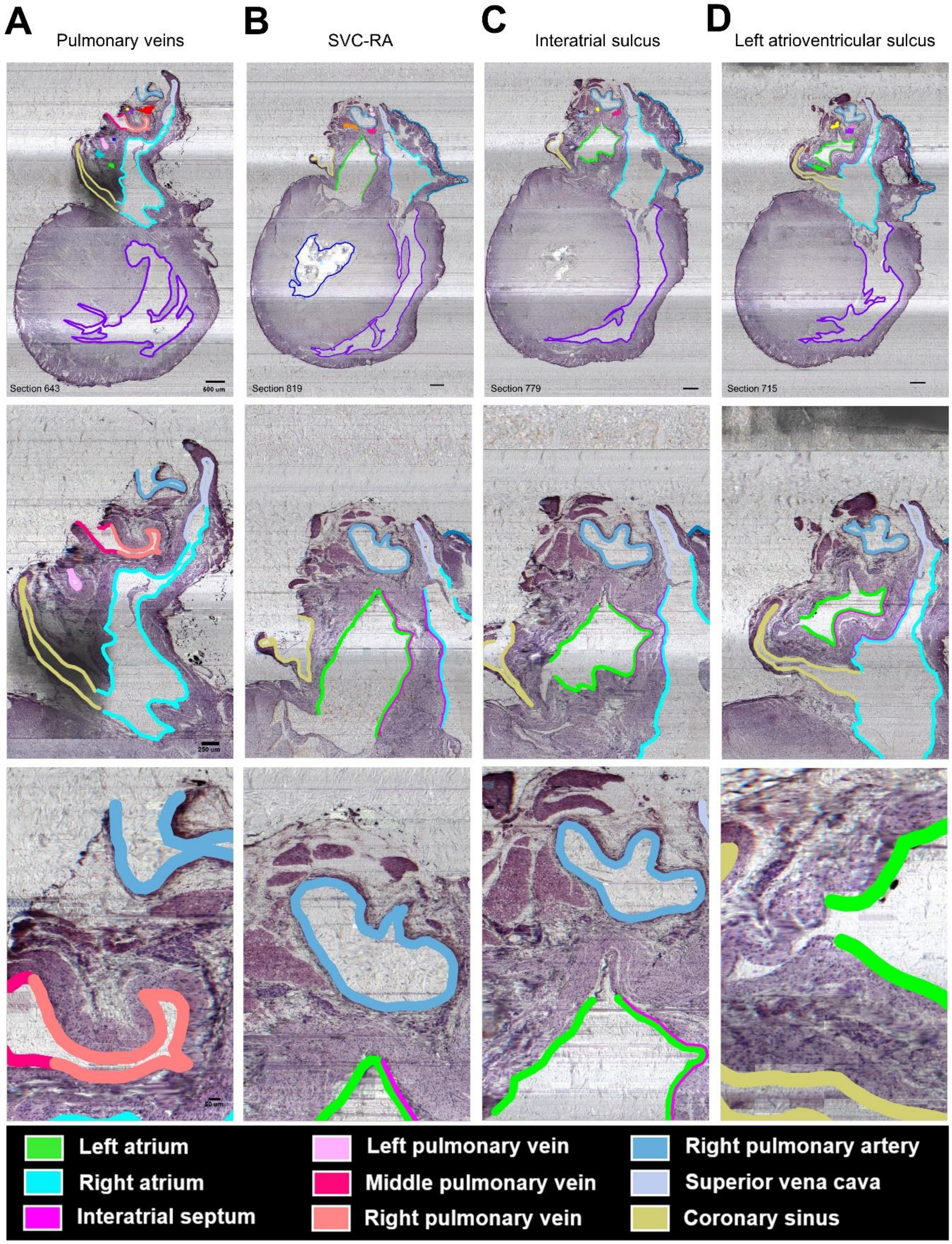

Figure S7: Histological sections of the male heart, related to Figure 3B, 5F, 7E, Video S4. (A-D) Histological sections from male heart M54-8 focusing on the pulmonary veins (A), superior root of the vena cava and the right atrium (B), interatrial sulcus (C), and left atrioventricular sulcus (D) at increased zoom levels from top to bottom.

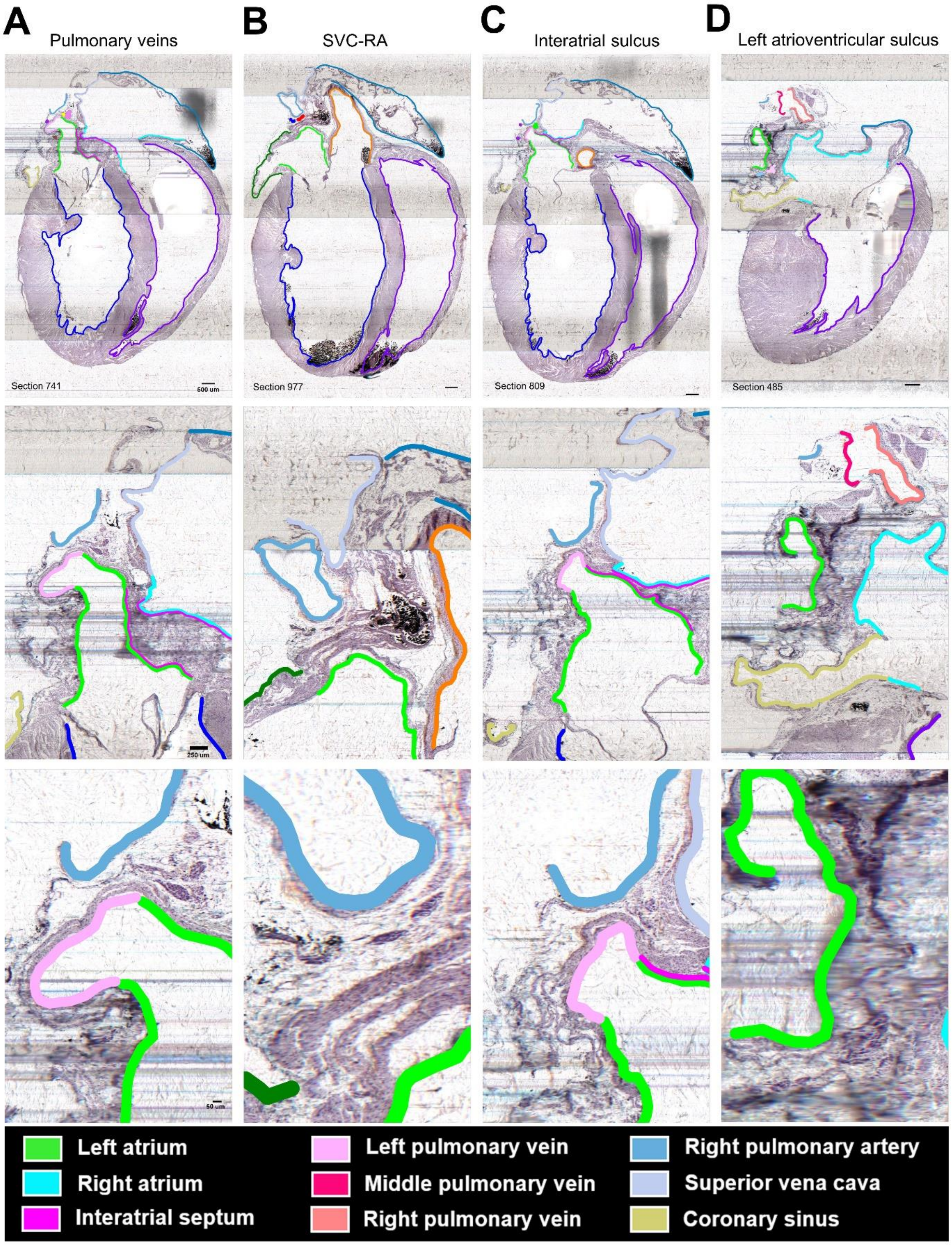

Figure S8: Histological sections of the female heart, related to Figure 7F, Video S5. (A-D) Histological sections from female heart F54-6 focusing on the pulmonary veins (A), superior root of the vena cava and the right atrium (B), interatrial sulcus (C), and left atrioventricular sulcus (D) at increased zoom levels from top to bottom.

Rat ID	Sex	Total neuron count	Average number of neurons (M vs. F)	Total number of sections in image stack	Total number of sections in image stack containing tissue	Sectioning plane
M54-5	Male	2,676	2,564	1,400	1,210	Parallel to long axis (coronal)
M54-8	Male	2,973		1,570	1,350	Parallel to long axis (coronal)
M54-9	Male	2,885		2,580	2,580	Parallel to short axis (transverse)
M54-10	Male	1,722		1,570	1,559	Parallel to long axis (sagittal)
F54-6	Female	1,858	1,581	1,650	1,494	Parallel to long axis (coronal)
F54-11	Female	1,468		1,380	1,344	Parallel to long axis (coronal)
F54-14	Female	1,418		2,460	2,448	Parallel to short axis (transverse)

Table S1, related to Figure 2: Details on the neuron count, number of sections in the image stack, and sectioning plane for each heart.
